# Neurological Complications of Biological Treatment of Psoriasis

**DOI:** 10.3390/life12010118

**Published:** 2022-01-14

**Authors:** Mateusz Kamil Ożóg, Beniamin Oskar Grabarek, Magdalena Wierzbik-Strońska, Magdalena Świder

**Affiliations:** 1 Department of Histology, Cytophysiology and Embryology, Faculty of Medicine, University of Technology in Katowice, 41-800 Zabrze, Poland; mateusz.ozog@wst.pl (M.K.O.); sekretariat@klinikamazan.pl (M.Ś.); 2 Department of Histology and Cells Pathology, Faculty of Medicine, Medical University of Silesia in Katowice, 41-800 Zabrze, Poland; 3 Department of Gynecology and Obstetrics, Faculty of Medicine, University of Technology in Katowice, 41-800 Zabrze, Poland; 4 Department of Gynecology and Obstetrics with Gynecologic Oncology, Ludwik Rydygier Memorial Specialized Hospital, 31-826 Kraków, Poland; 5 Department of Gynecology and Obstetrics, TOMMED Specjalisci od Zdrowia, 40-662 Katowice, Poland; 6 Faculty of Medicine, University of Technology in Katowice, 41-800 Zabrze, Poland; rektorat@wst.pl

**Keywords:** psoriasis, biological treatment, anticytokine therapy, tumor necrosis factor alpha, nervous system, side effects

## Abstract

In the available literature, little attention has been paid to the assessment of psoriasis and the biological therapy used for it and the nervous system. The purpose of this article is to discuss the relationship between psoriasis and the nervous system as well as to analyze the mechanisms that lead to neurological complications during anticytokine therapies in psoriasis. However, this connection requires further analysis. The use of biological drugs in psoriasis, although it yields positive therapeutic results, is not without numerous side effects. Serious neurological side effects of the therapy are most often visible with the use of anti-TNF-alpha, which is why patients should be monitored for their potential occurrence. Early detection of complications and rapid discontinuation of treatment with the drug may potentially increase the patient’s chances of a full recovery or improvement of his/her neurological condition. It also seems reasonable that, in the case of complications occurring during anti-TNF-alpha therapy, some of the drugs from other groups should be included in the therapy.

## 1. Introduction

Psoriasis is a noninfectious, chronic, systematic underlying autoimmune disease, which affects between 1–3% of the population [1]. During its course, there are inflammatory processes that are characterized by the presence of dermal symptoms as well as symptoms affecting the motor system. It generally occurs as plaque psoriasis located on the scalp, in the area of large joints, the area of the navel, and the loins. Other locations where changes occur include the face, palms, feet, and nails [2]. This kind of psoriasis concerns 85–90% of cases [3]. Other forms of psoriasis in which dermal changes occur are pustular, inverse, napkin, guttate, oral, and seborrheic-like, which affect approximately 10% of patients [4]. Palmoplantar involvement is usually associated with serious thickening of the skin and, in some rare cases, sterile pustules [5]. Psoriatic arthritis is an inflammatory condition of the joints which occurs in psoriasis. It affects 7–42% of the patients suffering from psoriasis [4], with 1–2% of patients exhibiting arthritis without the accompanying dermal symptoms [6]. The principal symptoms are inflammation of the spinal joints, neck pain, and inflammatory chest pain, as well as core symptoms [6,7]; 40–60% of the patients develop long-lasting joint complications [8]. In the available literature, little attention has been paid to the assessment of psoriasis and the biological therapy used for it and the nervous system. The purpose of this article was to discuss the relationship between psoriasis and the nervous system, as well as to analyze the mechanisms that lead to neurological complications during anticytokine therapies in psoriasis.

## 2. Etiopathogenesis of Psoriasis

**Figure 1:**
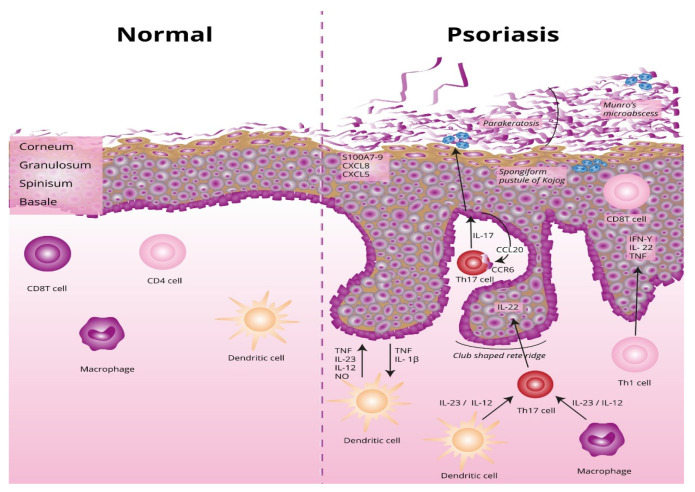
Differences between normal and lesional skin. TNF, tumor necrosis factor alpha; IL-23, interleukin 23; IL-12, interleukin 12; NO, nitric oxide; IL-1β, interleukin 1 beta; Th17, T helper 17; Th, T helper; CCL20, C-C motif chemokine ligand 20; CCR6, C-C motif chemokine receptor 6; CXCL5, C-X-C motif chemokine ligand 5; CXCL8, C-X-C motif chemokine ligand 8; S100A7-, psoriasin.

The underlying basis of psoriasis is skin inflammation (and, in the case of psoriatic arthritis, inflammation of the connective tissue which makes up the joints and the joint ligaments). The characteristic features of skin inflammation include hyperplasia of the epidermis, parakeratosis, and an inflammatory infiltration consisting of dendritic cells, macrophages, T-lymphocytes, and neutrophils [9]. Abnormalities in the skin’s immune response are responsible for the development of the inflammation. Endogenous risk signals cause the expression of the proinflammatory cytokines responsible for maintaining inflammation in the area of the skin. Figure 1 shows the differences between normal and lesional skin. Characteristic histological changes for cutaneous psoriasis can be observed. In diseased skin, psoriatic plaques have a compact and strongly thickened stratum corneum layer of the epidermis, no granular layer, an enlarged spinous layer, and an accumulation of neutrophils in the stratum corneum and the spinous layer of the epidermis. Within skin with psoriasis lesions, there is an accumulation of dendritic cells and macrophages, which are the source of inflammatory cytokines, which also contribute to the differentiation of naive T lymphocytes into the Th1 or Th17 subpopulations.

### 2.1. Parakeratosis

During the course of psoriasis, the principal symptoms are skin lesions that are reddish and brown-reddish flat protruding lumps of different sizes, covered with silvery-grey scales. They are created as a result of parakeratosis, which is a kind of keratinization characterized by preservation of the cell nucleus by the cells that make up the stratum corneum layer of the epidermis [10]. This inflammatory process [11] leads to replacing annular squames with nucleated cells.

### 2.2. Factors Precipitating the Development of Psoriasis and Stimulating the Development of Changes

The etiopathogenesis of psoriasis involves genetic factors (numerous genes significantly increasing the risk of contracting psoriasis have been described), environmental factors (such as stress, smoking, injuries, drugs, or bacterial flora), and immunological factors (an abnormal immune response from the T-lymphocytes, dendritic cells, and/or keratinocytes). Factors that evoke the development of changes are slight skin injuries, smoking, alcohol consumption, stress, conditions that cause severe hormonal changes (such as menstruation or menopause in women), some drugs, and infections [12].

#### 2.2.1. Genetic Factors

Psoriasis is certainly polygenic. Advancements in science have made significant progress in understanding the genetics of this type of dermatosis [12]. Nine different regions containing genes predisposing a person to the induction and development of the disease in question have been discovered, known as PSORS1–9, of which the most important role is played by the PSORS1 region. It is located on chromosome 6p21, where genes belonging to the major histocompatibility complex (MHC) are located, of which three (HLA-C, CCHCR1, and CDSN) are highly polymorphic. Numerous studies have confirmed their association with the development of psoriasis [12,13,14]. For example, Caputo et al. [14] confirmed that psoriasis is a very complex disease in which genetic, epigenetic and molecular factors play a key role. An important issue in the etiopathogenesis of the disease and the design of new drugs is to consider the fact of the mutual overlapping of many signaling pathways. Therefore, further research on psoriasis should consider the integration of large-scale epigenomic data and the three-dimensional organization of the genome into the so-called systems biology. This is possible thanks to the advancement of the omics era [14].

#### 2.2.2. Environmental Factors

The important role of environmental factors in the development of lesions underlines the complex nature of psoriasis. They play a key role in the initiation of the disease and its progress [15]. The environmental stressors that play a key role in the induction and development of psoriasis include noise, air pollution, improper diet, alcohol abuse, smoking, and drug addiction. Physiological stressors that contribute to the development of psoriasis are infections, primarily bacterial, and injuries, e.g., mechanical damage to the epidermis (the so-called Köbner effect) [16]. In turn, the psychological stressors include depression, incorrect social relations, family conflict, and professional problems. However, knowledge on this subject remains fragmented [17].

#### 2.2.3. Stress

In a study involving patients diagnosed with psoriasis, as many as 60% were deeply convinced that stress was the causative factor of their disease [18]. In other studies, it has been reported that psychological stress precedes the onset of the disease in 44% of psoriasis patients and initiates recurrent exacerbations in up to 88% of patients [19]. However, in some cases, no clear relationship between stress and disease exacerbations was found [20]. A marked increase in the severity of psoriasis is most often observed about 1 month after exposure to the stressors [21]. The vast majority of patients who report stress-induced psoriasis cite the feeling of cosmetic disfigurement and social stigmatization as the primary cause. The accompanying depressive disorders and reduced quality of life are especially pronounced in women [22].

#### 2.2.4. Pharmacotherapy of Comorbidities

Diseases comorbid with psoriasis cause an increased need for pharmacotherapy in a large group of patients.

Multiple preparations are believed to be possible psoriasis-worsening agents. The disease may appear de novo or be exacerbated [23].

Unfortunately, in clinical practice, it is usually difficult to link psoriasis outbreaks with medications. This is because the latency between treatment initiation and the onset of psoriatic skin lesions is different for many medications used for comorbid conditions. Moreover, with age, the phenomenon of polypragmasy increases significantly [23,24].

The most common pharmacological causes of psoriasis include: beta blockers, lithium salts, antismall drugs, angiotensin-converting enzyme inhibitors, and nonsteroidal anti-inflammatory drugs.

#### 2.2.5. Infections

Exacerbation of the course of psoriasis after an infection is associated with the so-called superantigens, which include bacterial, viral, and parasitic antigens. The similarity between the antigens of microorganisms and epidermal autoantigens may play an important role [25]. Among the infectious factors that can stimulate the formation of psoriasis lesions, the most important role is assigned to the Group A beta-hemolytic streptococci. Streptococcal infections of the upper respiratory tract are most often responsible for the acute appearance of psoriatic lesions in the droplet form of Type I psoriasis. Eruptions often appear quickly, usually within 2–4 weeks after bacterial contamination. Although the lesions are usually self-limiting, they may recur with subsequent streptococcal infections. Therefore, tonsillectomy may be a potential therapeutic option in patients with drug-resistant psoriasis accompanied by frequent episodes of tonsillitis [26,27].

#### 2.2.6. Nicotinism and Alcohol Consumption

Psoriasis patients often report a current or previous addiction to nicotine. Smoking may not only aggravate the course of existing psoriasis but may also increase the risk of de novo disease. A cross-sectional study showed that smokers of more than 20 cigarettes a day had twice the risk of developing severe psoriasis with a reduced likelihood of periods of remission than smokers of less than 10 cigarettes a day [28,29].

The relationship between alcohol consumption and psoriasis has long been controversial. Early research negated such connections, but observations carried out at the end of the 20th century showed significant relationships [30]. Alcohol may be a factor both initiating and intensifying inflammation by stimulating the multiplication of lymphocytes and the production of proinflammatory cytokines. In addition, alcohol can directly induce the proliferation of keratinocytes and increase the mRNA expression of genes related to this phenomenon through the production of proteins such as alpha5 integrin, cyclin D1, and the keratinocyte growth factor receptor (KGFR) [31,32,33]. To date, a limited number of studies have looked at the relationship between the severity of psoriasis and alcohol consumption; however, the observations made thus far seem to indicate the existence of such a relationship [30,31,32].

## 3. Biological Drugs Used in the Treatment of Psoriasis

According to the recommendations of the Polish Dermatological Society, the evaluation of the severity of the disease symptoms is conducted based on the Psoriasis Area and Severity Index (PASI), Body Surface Area (BSA), and the Dermatology Life Quality Index (DLQI) [34]. Topical treatment is the only recommended treatment method for patients with mild psoriasis, who score no more than 10 points on the abovementioned scales. The most used topical treatments are based on vitamin D derivatives and steroids [35]. Patients with diagnosed plaque psoriasis of moderate or acute severity, and those with arthropathic psoriasis who do not react to traditional treatment qualify for biological treatment [34,36]. Although the clinical condition of 70–89% of patients [3] allows for the use of topical treatment, which has less severe side effects, these preparations require time-consuming application [34]. Classical systemic therapies include numerous drugs containing methotrexate, cyclosporine A, and retinoids [36,37]. The current work aimed to focus on the neurological side effects of biological drugs used in the treatment of psoriasis in connection with the increasingly common occurrence of psoriasis within the population [38], as well as the increasing availability of biological treatment. The biological drugs currently used in the treatment of psoriasis can be divided into several groups depending on their mechanism of function (Table 1).

## 4. Connections between Psoriasis and the Nervous System

In the literature on this subject, many connections have been described which testify to a significant increase in the risk of neurological diseases in the course of psoriasis. Patients suffering from psoriasis have a much higher risk of stroke (mild psoriasis: HR = 1.20, 95% CI: 1.06–1.35; severe psoriasis: HR = 1.70, 95% CI: 1.18–2.43) [44], multiple sclerosis (OR = 1.521, 95% CI: 1.01–2.29; *p* = 0.04) [45], epilepsy (OR = 3.8, 95% CI: 3.6–4.0) [46], migraine [47] (OR = 1.8, 95% CI: 1.55–2.09), and Parkinson’s disease (HR = 1.091, 95% CI: 1.029–1.115) [48]. There have also been numerous cases describing patients who suffer from both psoriasis and myasthenia gravis [49,50,51]; however, this connection requires further analysis.

An increase in the incidence of neurological diseases can be connected with an increase in the concentration of cytokines participating in the pathomechanism of psoriasis [52] (Table 2).

## 5. Neurological Complications and Adverse Reactions of the Biological Treatment of Psoriasis

Serious neurological complications of the biological treatment of psoriasis, although not very common, constitute a serious clinical problem. Usually, as soon as they appear, the therapy should be discontinued. However, even though the drug is no longer administered and treatment is implemented against a given complication, the patient does not always return to his/her original state as far as neurological condition [58,59,60,61,62,63,64,65,66,67,68,69,70]. Table 3 summarizes adverse reactions of the biological treatment of psoriasis, including specific neurological complications or side effects for each biological drug.

In the following sections, we describe the neurological complications associated with the use of biologics for psoriasis.

### 5.1. TNF-Alpha Inhibitors

TNF-alpha inhibitors constitute a group of drugs that are well-tolerated by patients. Typical side effects include headaches, reactions in the area of administration, rashes, anemia, an increase in transaminases, coughing, nausea, and stomachaches. There is also an increased risk of infectious diseases in this group of patients, especially infections of the upper respiratory tract, sinusitis, and diarrhea [71,72,73,74].

In accordance with the data submitted to the Food and Drug Administration Adverse Event Reporting System, more than half the patients treated with this group of drugs develop serious neurological complications within the period of a 10-year observation [75]. The most common complications included demyelinating diseases of the CNS and the spinal cord, optic neuritis, peripheral neuropathy, and facial palsy [75,76]. Other complications that have been reported included transverse myelitis, Guillain–Barré syndrome, myasthenia gravis, Parkinson’s disease, cancers of the CNS, and infectious diseases of the CNS [77]. Despite the fact that some works have suggested a relationship between treatment with TNF-alpha and an increased risk of neurovascular diseases of the CNS, it has been shown that in this population of patients, this risk is lower than approximately 12% [78]

#### 5.1.1. Demyelinating Diseases of the CNS and the Spinal Cord

A demyelinating disease of the CNS and the spinal cord is the most common neurological complication occurring during anti-TNF-alpha treatment [39]. These complications most often occur in patients who are 51 years old on average, and in women [79,80]. Typical clinical symptoms are mental confusion, paresthesia, speech impediments, cognitive impairments, and numerous impairments of motor activity (apraxia, paralysis of the limbs, or hemiparesis) [44]. Typical changes in the MRI in T1 and T2 sequences of the central nervous system are newly created demyelinating foci [81]. The diagnosis of a demyelinating disease of the CNS is presented in Table 4. The most common method of treatment in these cases is methylprednisolone and other intravenous immunoglobulin preparations. Various authors have also pointed out cases in which different methods of treating IFN-beta were used. Stopping anti-TNF-alpha therapy and using pulse MP and IVIG allows for the recovery of approximately 35–37% of patients [79,80].

#### 5.1.2. Optic Neuritis

Optic neuritis is the second most common neurological complication occurring during anti-TNF-alpha treatment [39]. This complication most often affects patients who are 40 years old on average, and women [82].

Apart from changes in the eyes in this group of patients, changes in the MRI may also be present, such as optic disc swelling, as well as demyelinating changes in the CNS [82]. Standard treatment includes administering pulse MP, oral administration of glucocorticosteroids, and INF. Only some patients attain full recovery [83].

**Table 4:** Diagnosis of peripheral neuropathy in the course of anti-TNF-alpha treatment [84,85,86,87,88].

Test	Results
Neurological test	Motor disorder as the neuropathy that develops is predominantly motor in nature
Electromyography	Demyelination
Cerebrospinal fluid testing	Often increased total protein concentration
Nerve biopsy	Demyelination
Peripheral nerve ultrasound	Nerve cross-sectional areas in nerves responsible for a given area of the body

#### 5.1.3. Peripheral Neuropathy

Another common complication in this group of patients is peripheral neuropathy [75]. It most often occurs in patients who are approximately 50 years old, and in men [84]. In the literature on the subject, many different locations and forms of peripheral neuropathy may be encountered in this group of patients. The most common form is demyelinating polyradiculopathy (both motor and sensory), as well as inflammation of a single nerve root [84,85].

Due to an increased risk of Type II diabetes and metabolic syndrome in patients with psoriasis [86,87], extra care must be taken when qualifying patients who have been diagnosed with these metabolic disorders for anti-TNF-alpha treatment. This group of patients is particularly susceptible to the development of diabetic polyneuropathy [88].

The diagnosis of peripheral neuropathy in patients treated with immunoglobulins, with a particular emphasis on patients treated with anti-TNF-alpha, is presented in Table 4. In an EMG, the typical pathology is demyelination of the nerve fibers [84,85], where, in most patients, a higher protein concentration can be observed in the cerebrospinal fluid (lumbar puncture is a standard procedure in the diagnosis of polyneuropathy) [84]. Typical treatment is based on discontinuing anti-TNF-alpha therapy, as well as IVIG. In most cases, full recovery is achieved [84,85]. Despite the fact that the authors did not point out specific alternatives as far as further treatment of psoriasis, it seems reasonable to replace TNF-alpha inhibitors with drugs from other groups.

The diagnosis of peripheral neuropathy in patients treated with immunoglobulins is summarized in Table 4.

#### 5.1.4. Facial Palsy

Facial palsy during anti-TNF-alpha therapy is a less frequent complication than the ones described above; however, it still affects a significant number of patients [75]. In the cases described in the literature, the patients had symptoms of central damage to the optic nerve. In the MRI scans, swelling of the trunk of the facial nerve is visible. Typical treatment involves discontinuation of the anti-TNF-alpha therapy, as well as glucocorticosteroids. The chances of full recovery are good [89,90,91].

#### 5.1.5. Myasthenia Gravis

Despite evidence of potential effectiveness of treating myasthenia gravis with the use of etanercept [92,93], several cases of a diagnosis of myasthenia in the course of anti-TNF-alpha treatment have been described [58,59]. The patients developed antibodies against acetylcholine receptors in the blood serum. The development of symptoms of myasthenia gravis occurred within several months, quickly leading to muscle fatigue and problems with breathing. In these patients, therapy with TNF-alpha inhibitors was replaced by glucocorticosteroids. The expected improvement was achieved. Therefore, in this group of patients, it seems that TNF-alpha inhibitors should not be used [94,95].

### 5.2. Ustekinumab, an IL-12 and IL-23 Inhibitor

Typical side effects of ustekinumab include headaches, joint pain, nausea, fever, stomachaches, and weakness. There is also an increased risk of infection in this group of patients [96]. In terms of neurological complications, ustekinumab should be deemed as a relatively safe drug. In Phase III clinical testing conducted on a group of 3758 patients suffering from psoriasis, only a single case of posterior reversible encephalopathy syndrome was described [97]. The literature also describes the cases of a patient treated for psoriatic arthritis and two patients treated for Crohn’s disease who also developed complications [98,99]. The posterior reversible encephalopathy syndrome is a disease syndrome exhibiting encephalopathy, mainly as a quantitative and qualitative disturbance of consciousness, and hypertension. Patients often experience convulsions, nausea, vomiting, and headaches. During the course of posterior reversible encephalopathy syndrome (PRES), different focal disorders may occur; in extreme cases there can be life-threatening epilepsies, cerebral syndrome, hemiplegia, and coma. The symptoms described above are a direct result of vasogenic cerebral edema [100].

Lab, electrophysiological, and imaging tests useful in the diagnosis of this syndrome are presented in the Table 5.

Presently, there is no targeted treatment of this syndrome; instead, symptomatic treatment is used. It is believed that lowering the patient’s blood pressure to normal levels is of key importance, using aggressive hypotensive treatment, such as nitroglycerine, sodium nitroprusside, labetalol, urapidil, and nicardipine intravenously [101]. In all the cases described in literature after discontinued ustekinumab as well as antihypertension therapy, the patient’s condition stabilized [98,99]. In such cases, it is recommended to use a drug from a different group.

There has also been a case described in the literature of a patient where, after administration of ustekinumab, myasthenia gravis developed. This patient had been previously treated with etanercept, which was discontinued due to inadequate long-term control of psoriatic arthritis symptoms. After 6 months of ustekinumab along with methotrexate, a thymoma was discovered; 3 months later, the full symptoms of myasthenia gravis developed. After a more thorough analysis of the patient’s medical history, it turned out that initial symptoms which may have been counted as a manifestation of myasthenia gravis began developing 6 years prior. After discontinuation of biological treatment, increasing the methotrexate dose, and adding prednisone, a full reduction of the symptoms could be seen after 3 months. Due to a short follow-up, there is no information regarding the continuation of psoriasis treatment [102,103].

### 5.3. IL-23 Inhibitors

The typical side effects of using drugs from this group include fatigue, headaches, joint pain, and local reactions in the place of administration. An increased risk of infection has also been observed [104,105,106]. In the case of IL-23 inhibitors, Phase III clinical tests have not shown any neurological complications [104,105,106]. In the literature, there is only a description of a single case of sensorimotor axonal polyneuropathy. After unsuccessful treatment with methotrexate, the patient qualified for therapy with guselkumab. However, after 1 month of treatment, the patient exhibited the first neurological symptoms, then after 10 months, due to continued development of the symptoms of polyneuropathy, the treatment was discontinued. After discontinuation of the treatment, the patient’s condition began to improve; however, after more than 1 year of observation, the symptoms of polyneuropathy did not completely disappear, although electromyography showed re-innervation changes. After half a year, apremilast was added to the therapy, which, due to its ineffectiveness, was later replaced by secukinumab [107].

### 5.4. IL-17 Inhibitors

The typical side effects of therapy with IL-17 inhibitors include throat ache, joint pain, and headaches. This group of patients also has a much higher risk of infection, especially infection of the upper respiratory tracts [108,109,110]. In Phase III testing, no neurological complications were seen. At present, the literature has not described any cases. Due to the length of the clinical tests and the size of the groups, it may be assumed that using IL-17 inhibitors is most likely safe on the neurological level [110].

### 5.5. Abatacept, a T-Lymphocyte Inhibitor

The typical side effects of using abatacept include diarrhea and vomiting. Due to the immunosuppressive activity of abatacept, the risk of infection is also higher. Phase III tests did not show any neurological complications [111,112]. The literature shows one case of peripheral polyneuropathy in an 8-year-old girl treated with abatacept due to juvenile idiopathic arthritis. Abatacept was used due to previous treatment with etanercept having had no effect. Within a few months, peripheral polyneuropathy developed. After discontinuation of the drug and introducing methylprednisolone, full recovery was achieved [113].

## 6. Possible Explanations of the Mechanism Leading to Complications, Including the Connection between Psoriasis and the Nervous System

Due to the rarity of neurological complications within the course of therapy with ustekinumab, abatacept, and IL-23 inhibitors, the mechanism remains unknown. In the case of TNF-alpha inhibitors, there are several theories that attempt to explain their incidence. Due to its pleiotropic impact on the cells of the CNS, TNF-alpha may both stimulate and inhibit inflammatory processes in the area of the nerve tissue. These effects depend on the type of the stimulated receptor and the types of cells that make up the nerve tissue [114]. TNF-alpha is responsible for the suppression of the production of encephalitogenic lymphocytes Th1 and Th17, while stimulating the inflammatory infiltration of the nervous system through stimulating the production of other proinflammatory cytokines [115]. In addition, it is responsible for stimulating the conversion of Th lymphocytes into Th-17 lymphocytes through stimulating monocytes to produce IL-6 and IL-1beta. TNF-alpha is also necessary in the process of remyelination and it promotes the proliferation of oligodendrocyte precursor cells [115,116].

There are several possible explanations of the very frequent neurological side effects within the course of anti-TNF-alpha therapy. The first is that the decrease in the concentration of active TNF-alpha in the area of the nerve cell may lead to inhibition of the molecular cascades connected with the TNRF receptors whose stimulation is necessary for the proliferation of oligodendrocytes and remyelination [80]. The second possibility is an abnormality of the immune defense mechanism of the nerve tissue resulting from inhibition of the activity of TNF-alpha, which may lead to the activation of latent infection of the CNS [117]. The basic argument against these theories is the fact that the available anti-TNF-alpha drugs do not cross the blood–brain barrier [118].

Another theory that may explain the common incidence of neurological complications within the course of therapy with this drug group is the local increase in the concentration of TNF-alpha in the brain tissue (“sponge effect”). While the concentration of TNF-alpha during the therapy decreases in the peripheral tissues, it remains at a relatively high level in the area of the nerve tissue [117]. A lack of balance between the concentration of TNF-alpha in the peripheral tissue and the nerve tissue may cause increased infiltration of the lymphocytes which are directly responsible for damage in the area of the nerve tissue through the release of cytokines [117], which is a key phenomenon of yet another theory. Another potential phenomenon that may occur in this group of patients is the increased serum neutralization capacity of TNF-α, which leads to a decrease in the activity of TNF-alpha in the area of the nerve tissue and, as a result, a lack of activation of remyelination [83], for which the appropriate concentration of TNF-alpha is required (such an effect was discovered in patients with multiple sclerosis) [115].

## 7. Conclusions

Using biological drugs in psoriasis, although it yields positive therapeutic results, is not without numerous side effects. In some cases, their incidence is connected to the discontinuation of therapy. Serious neurological side effects of the therapy are most often visible with the use of anti-TNF-alpha, which is why patients should be monitored for their potential incidence. Early detection of complications and rapid discontinuation of treatment with the drug may potentially increase the patient’s chances of a full recovery or an improvement in his/her neurological condition. Particular care must also be emphasized regarding anti-TNF-alpha therapy for patients whose family medical history shows the incidence of neurodegenerative diseases due to a lack of certainty as far as the mechanism of development of these complications. As far as the incidence of neurological complications, other drug groups seem relatively safe, e.g., ustekinumab, abatacept, IL-23, and IL-17 inhibitors. In these cases, serious neurological complications occur in relatively few patients. However, the risk of their incidence is not to be overlooked, and patients treated with them should also be monitored during the treatment. It also seems reasonable that, in case of the incidence of complications during anti-TNF-alpha therapy, some of the drugs from other groups should be included in the therapy.

## Figures and Tables

**Table 1:** Classification of antipsoriatic drugs by their biological influence.

Group	Marketed Formulations	Biological Influence of the Drug
TNF-alpha inhibitors	Certolizumab pegol Etanercept Adalimumab Infliximab Golimumab	Patients with psoriasis exhibit an excessive production of TNF-alpha in the skin as well as the joints. This is a proinflammatory cytokine that acts through by stimulating the release of numerous proinflammatory factors, which ultimately leads to inflammatory infiltration in the area of the skin. Through the inhibition of this cytokine, the inflammation in the skin area is reduced [39].
IL-12 and IL-23 inhibitors	Ustekinumab	IL-12 and IL-23 are constructed from a common p40 subunit, which ustekinumab acts against. IL-12 stimulates NK (natural killer) cells and differentiation of CD4^+^ T cells towards the Th1 phenotype. Ustekinumab, if it is unable to attach to IL-12 or IL-23, which are attached to the IL-12Rβ1 receptors on the cell surface, does not affect complement activity and is not involved in antibody-mediated cytotoxicity of the receptor cells. Ustekinumab can exert its clinical effects in psoriasis and psoriatic arthritis by disrupting the Th1 and Th17 cytokine pathways that play a key role in the pathology of these diseases [40].
IL-23 inhibitors	Tildrakizumab Risankizumab Guselkumab	Recent studies indicated that IL-23 is the most important cytokine in the pathogenesis of psoriasis, as it induces the differentiation of naïve T lymphocytes towards the Th17 phenotype and thus to the formation of psoriatic plaque. The newest p19 inhibitor of IL-23 is risankizumab, which has a good safety profile, less frequent use, and suitable efficacy in severe psoriasis [41].
IL-17 inhibitors	secukinumab brodalumab ixekizumab	IL-17 is a cytokine that causes an increase in the expression of factors such as TNF-alpha, stimulating the development of inflammatory infiltration. Blocking IL-17 causes a significant reduction in infiltration [40].
T-lymphocyte inhibitors	Abatacept	In the area of skin changes, there are numerous T-lymphocytes with impaired function. This causes the stimulation of an improper inflammatory reaction [42]. The cytotoxic activity of abatacept on T-lymphocytes causes a decrease in their population, resulting in a reduction of the inflammation in the area of psoriatic changes [43].

**Table 2:** Impact of cytokines involved in the pathomechanism of psoriasis on the nerve tissue.

Cytokine	Impact on Nerve Tissue
TNFα	TNF-alpha receptors are present on the surface of neurons as well as astrocytes and microglia. The stimulation of these receptors causes the activation of cascades leading to cell apoptosis and changes as far as the expression of genes responsible for the survival of a cell [53]. The exposure of nerve tissue to a high concentration of this cytokine results in the decomposition of microglia and a loss of neurons [54], which can lead to neurodegenerative diseases.
IL-12	The exposure of the nervous system to a high concentration of IL-12 may induce the development of neurodegenerative diseases by inducing neuron apoptosis and stimulation of the proliferation of astrocytes [55].
IL-17	A high concentration of this cytokine may cause the activation of the glia and the infiltration of the CNS by proinflammatory cells [56].
IL-23	A high concentration of this cytokine may cause the activation of the glia and the infiltration of the CNS by proinflammatory cells [57].

TNF-α, tumor necrosis factor alpha; IL-12, interleukin 12; IL-17, interleukin 17; IL-23, interleukin 23.

**Table 3:** Specific neurological complications and side effects related to biological therapy for psoriasis.

Group	Marketed Formulations	Specific Neurological Complications or Side Effects			
		Very Often	Often	Seldom	Rarely
TNF-alpha inhibitors	Certolizumab pegol [58]	-	Headache (including migraine), sensory disturbance	Mental disorders (anxiety and mood disorders, peripheral neuropathies, dizziness, tremors	Convulsions, inflammation of the cranial nerve, impaired coordination or balance, multiple sclerosis, Guillain–Barré syndrome
	Etanercept [59]	Headache, demyelinating polyneuropathy, and multifocal motor neuropathy	-	Cases of CNS demyelinating syndromes (e.g., multiple sclerosis) or limited demyelinating syndromes (e.g., optic neuritis and transverse myelitis); cases of peripheral demyelinating polyneuropathy, including Guillain–Barré syndrome, chronic inflammatory demyelinating polyneuropathy	-
	Adalimumab [60]	Headache	Mood changes, including depression and anxiety; insomnia; paraesthesia (including hypoesthesia); migraine compression of the nerve root	Stroke, muscle tremors, neuropathy	Multiple sclerosis, demyelinating disorders (e.g., optic neuritis, Guillain–Barré syndrome)
	Infliximab [61]	Headache	Depression, insomnia, vertigo and post-hypoesthesia, hypoesthesia, paresthesia	Amnesia, agitation, confusion, seizure, neuropathy	Sleepiness, nervousness. apathy, transverse myelitis, central nervous system demyelinating diseases (multiple sclerosis-like diseases and optic neuritis), peripheral demyelinating diseases (such as Guillain–Barré syndrome, chronic inflammatory demyelinating polyneuropathy, and multifocal motor neuropathy), cerebrovascular accidents in close temporal association with the infusion
	Golimumab [62]	-	Dizziness, headache, paresthesia	Depression, insomnia, dizziness, headache, paresthesia	Balance disorders, demyelinating diseases (central and peripheral nervous system), taste disturbances
IL-12/23 inhibitors	Ustekinumab [63]	-	Dizziness, headache	Depression, facial nerve palsy	-
IL-23 inhibitors	Tildrakizumab [64]	-	Headache	-	-
	Risankizumab [65]	-	Headache	-	
	Guselkumab [66]	-	Headache	-	-
IL-17 inhibitors	Secukinumab [67]	-	Headache	-	
	Brodalumab [68]	-	Headache		-
	Ixekizumab [69]	-	-	-	-
T-lymphocyte inhibitors	Abatacept [70]	-	Headache, dizziness	Depression, anxiety, sleep disturbances (including insomnia), migraines, paresthesia	-

**Table 5:** Posterior reversible encephalopathy syndrome diagnosis [99,100].

Diagnostic Tool	Finding
Laboratory data	Hypomagnesemia
	Lactate dehydrogenase ↑
	Liver function parameters ↑
	Creatinine ↑
	Albumin ↓
Cerebrospinal fluid	Albumin ↑
	Albuminocytologic dissociation
EEG	Diffuse theta slowing
	Delta slowing
	Rhythmic delta activity
	Sharp-slow wave activity
	Periodic lateralizing epileptiform discharges
	Diffuse or focal (symmetric) slowing of background activities
CT and MRI	Vasogenic edema
	Watershed distribution
	Parieto-occipital pattern
	Frontal and temporal lobe involvement
	Subcortical white matter lesions
	Bilateral, frequently symmetric distribution
	Hyperintense T2-weighted and FLAIR sequences
	Iso-, hypo-, or hyperintense lesions on DWI
	Facultative contrast enhancement
	Microbleeds, intracerebral hemorrhage possible
	Increased or decreased ADC values depending on or indicating the (ir) reversibility of lesions
Angiography	Vasoconstriction, vasospasm (diffuse or focal)

↑—increase; ↓—decrease; EEG, electroencephalogram; CT, computed tomography; MRI, magnetic resonance imaging; FLAIR, fluid-attenuated inversion recovery; DWI, diffusion-weighted imaging; ADC, apparent diffusion coefficient.

## Data Availability

All data generated or analyzed during this study are included in this published article.
